# Suicide and the psychiatrist: Commentary on… Effects of patient suicide on psychiatrists

**DOI:** 10.1192/bjb.2019.53

**Published:** 2019-10

**Authors:** Allan House

**Affiliations:** 1 Leeds Institute of Health Sciences, School of Medicine, University of Leeds, UK

**Keywords:** Suicide, psychiatrist

## Abstract

Research into patient suicide indicates that it has an impact on the psychiatrists involved, but leaves a number of unanswered questions about which elements of the experience are most likely to cause problems, who is most at risk, what is the clinical or professional significance of any effect on the psychiatrist and how other professionals are affected. Despite these uncertainties, it is clear that a response is needed, with three bodies responsible in different ways for coordinating one: the relevant mental health trust, as employer; the Royal College of Psychiatrists, as the professional representative body; and the National Confidential Inquiry into Suicide and Safety in Mental Health, as mediator of social and professional impact.

Something like one in four of all those who die by suicide in the UK have had recent contact with mental health services, so it is not surprising that many psychiatrists will have close experience during their career of this worst of all outcomes. Neither is it surprising that there are emotional and practical consequences of such an experience for the psychiatrists involved.^1^ A paper by Gibbons and colleagues in this issue^2^ describes the findings from a survey of psychiatrists about their experiences of patient suicide and serves as a useful reminder of the importance of this aspect of psychiatric practice; at the same time the results raise a number of questions about the specifics that lie behind these apparently self-evident truths.

## The nature of the experience of patient suicide

What we are considering here is the idea of patient suicide as a life event for the psychiatrist. That is, it is an occurrence that directly impinges on the individual practitioner and has the potential to provoke a response that may be transient and unremarkable or may reach the level of intensity and duration that amounts to a disorder. What exactly is the nature of the exposure being considered?

In life-events terms, patient suicide is a complex event. That is, it has a number of more-or-less essentially interrelated features. There is the death itself and its immediate circumstances; inevitably, there is a formal inquiry (or more than one) in which the psychiatrist is likely to be asked to account for their own involvement; colleagues and close others make their own responses; finally, there is likely to be media coverage.

In relation to the first element of this complex – the death itself – the mention of post-traumatic stress disorder (PTSD) raises a question about level of exposure and proximity to the event. The UK's National Confidential Inquiry into Suicide and Safety in Mental Health (NCISH) has defined recent service contact as being within the 12 months before death;^3^ the survey reported here asked about suicide of a patient the psychiatrist had ‘been working with’, which suggests, without being specific, a more immediate connection (proximity). Surely the death of an in-patient in which staff are directly involved with attempts at resuscitation will have different effects from the death of somebody seen as an out-patient weeks or months before it occurred? The question is – should we, in the name of efficiency, focus more on the effects of some suicides (or the suicides of some people) than of others or should we accept that all suicides merit a staff-support response?

Life-events research tells us that external events are not just shocking in some non-specific way, they have a meaning that shapes the response we make – loss causing depression, threat causing anxiety and so on. While it is not difficult to understand the patient's death itself as uncomplicatedly negative – with perhaps an element of loss, threat and (under certain circumstances) the existential threat that constitutes trauma – the other elements of the exposure outlined above are less clear cut. They may be negative – critical, blaming, inducing guilt – or they may be positive – conveying support, affirmation, exoneration. It is difficult to see that these other aspects of the experience can be avoided, so the question is – can they be moderated either by direct influence on other parties or by support for the involved psychiatrist?

## Who is most at risk?

Life-events research has produced a certain amount of ambiguity about the cumulative effect of multiple events. If one event is not severe enough to cause an emotional problem, what about several subthreshold events, are they additive? One of the more surprising (to me) findings from the present study^1^ relates to the number of respondents who had experience of multiple patient suicides. Is this a question of age (years of exposure)? Area of work? What difference does it make? Is this a situation where multiple exposure sensitises or desensitises? This sort of accumulated experience of patient suicide is not likely to be picked up by suicide review, where the focus is on the patient and their care. Rather, it raises a question about staff review and how uncommon but important events can be monitored over time.

## What exactly are the effects?

One of the inevitable weaknesses of questionnaire surveys is that they leave certain questions underexplored, and in this case it is difficult to judge exactly how severe the reported responses were. Clearly, the emotional effect of such an event can be significant, but in clinical terms even in a selected sample only a very small number took time off work or thought they were ill. It is not clear how many respondents sought professional help for their mental health, although about a quarter said that some form of counselling or therapy would be a good idea. In conventional psychiatric terms most of this would be described as no more than mild or moderate disturbance and would be unlikely to be accepted for treatment following referral to the average community mental health team. Is this a matter for occupational health or for the marshalling of personal resources? The majority of respondents in this study acted as if they thought the latter, whereas the authors conclude that the answer ought to be more towards the former.

An intriguing question not raised by the authors is the degree to which the response to patient suicide might have a positive dimension. There is a substantial literature on personal growth consequent upon adversity, and another possibility is raised by the observation that women reported a greater sense of responsibility and effect on their clinical confidence. There is evidence from other areas of medicine that female doctors tend to practise differently, with better outcomes,^4^ raising the question of whether the problem in medicine is female diffidence or male overconfidence. A worried sense of responsibility and questioning of our competence may be stressful, but it is not necessarily bad for our patients if it leads^1,5^ to more vigilance and desire for involving others when managing patients perceived to be at risk of suicide.

## What about other mental health professionals?

Most mental health practice involves multidisciplinary teams – ward teams, crisis teams, home treatment teams, community mental health teams. Even in out-patient services, the patient who sees a psychiatrist is quite likely to have contact also with a community psychiatric nurse or other professional. It is a striking feature of the present study – and a number of the others cited – that these other professionals are not mentioned. One wonders whether some part of the psychiatrist's involvement had been to contribute to team discussions about the implications of a patient suicide, or to offer support to a non-medical colleague? Certainly, there is no reason to believe that doctors alone are vulnerable to the stresses of clinical work.^6^

As a mild digression, it is interesting to note how little literature there is on the effects of patient suicide in clinical psychology and improving access to psychological therapies (IAPT). These services have a reputation for reluctance to take on patients perceived to be at risk of suicide, but even so it must be the case that some suicides occur while the patient is ‘working with’ or has had recent contact with the relevant professionals. For example, the wider impact of suicide is discussed in a recent British Psychological Society publication,^7^ but the effect on professionals is not covered. The implication is that, as in psychiatry, the expectation is for individual practitioners to manage for themselves.

## A final question: what are the implications?

Gibbons and colleagues, no doubt wishing to avoid wandering too far from their data, make few recommendations about what should happen next. Nonetheless, there are implications of their findings.

First, patient suicide is a complex event with a number of components that represent relatively predictable challenges for the mental health professional. The most common perceived needs in the present survey were for instrumental and informational support in facing these challenges. It is surely the employing organisation's responsibility to provide such support, organised via the medical director. It would be helpful if the Royal College of Psychiatrists were to provide practical guidance. The NCISH could also have a role here. Researchers are expected to indicate to research ethics committees how they will ensure that participants from whom they collect information are aware of how to access help; the NCISH could act as a useful conduit for independent (from the employing trust) guidance to psychiatrists faced with the death of a patient on whom they are providing data.

Second, the ‘exposure’ in patient suicide varies in intensity and proximity, and the emotional and social impact also varies. This argues against, as does much other work in trauma response, a global approach to preventive intervention at an individual level. Follow-up of staff at (say) 6 months after a patient suicide could readily be incorporated into staff review and support – especially for those in high-risk subspecialties or with multiple experiences of patient suicide.

Finally, we should embrace multidisciplinarity in this as in other areas of practice. It would be a useful collaborative exercise for the College to convene a working group with other official bodies, to include at least the British Psychological Society and Royal College of Nursing, with the aim of producing a common set of guidelines on staff support after patient suicide – a small but important part of the larger question of health and well-being in the NHS workforce.

## About the author

**Allan House** is Professor of Liaison Psychiatry at the Leeds Institute of Health Sciences, School of Medicine, University of Leeds, UK.
